# The Obstetric Memoirs and Contributions of James Y. Simpson, M. D.

**Published:** 1856-12

**Authors:** 


### BOOK NOTICES.

*The Obstetric Memoirs and Contributions of* JAMES Y. SIMPSON, M.D. F.R.S.E. Prof. of Midwifery in the University of Edinburgh, &c. Edited by W. O. PRIESTLY, M.D. Edinburgh, and HORATIO R. STORER, M.D. of Boston, U.S. one of the Physicians to the Lying-in-Hospital, &c. Vol. II. Philadelphia, J. B. Lippincott & Co. 1856.

This is a volume of seven hundred and thirty-three pages, printed on fair type and paper, but very coarsely bound in cloth, without being sufficiently trimmed to cut the leaves apart. It contains Chapters or Essays, by Prof. Simpson, on the following highly-interesting subjects, viz.:—

*First*—Pathology of the Puerperal State.

*Second*—Physiology and Pathology of the Products of Conception.

*Third*—Pathology of Infancy and Childhood.

*Fourth*—Anæsthesia in Surgery.

*Fifth*—Anæsthesia in Midwifery.

*Sixth*—On the Nature and Powers of Various Anæsthetic Agents.

*Seventh*—On Local Anæsthesia.

The matter contained in the last four sections had been previously extensively re-published in this country. The whole volume, however, will be found extremely valuable to the practitioner, being replete with interesting facts drawn from observation and experience, and should consequently have a place in every medical library.

It is for sale at D. B. Cooke & Co. 112 Lake Street, Chicago.

